# Concern about: Echinacoside exerts anti-tumor activity via the miR-503-3p/TGF-β1/Smad aixs in liver cancer

**DOI:** 10.1186/s12935-021-02311-1

**Published:** 2021-11-22

**Authors:** Jiong Lin

**Affiliations:** Department of Oncology, Affiliated Hospital of Guangdong Medical University, No. 27 South of Ren Min Road, Zhanjiang, 524200 Guangdong China

**Keywords:** Echinacoside, Liver cancer, Invasion, Apoptosis, Proliferation

## Abstract

We concentrated on a paper in Cancer Cell International “Echinacoside exerts anti-tumor activity via the miR-503-3p/TGF-β1/Smad aixs in liver cancer”. Echinacoside may be a safe and effective anti-tumor agent for the treatment of liver cancer. However, some problems in this paper made us confused.

To the editor,

With high enthusiasm, we read a paper “Echinacoside exerts anti-tumor activity via the miR-503-3p/TGF-β1/Smad aixs in liver cancer” published in Cancer Cell International [1]. Li et al. revealed that echinacoside could retard cell proliferation, migration, and invasion, and induce apoptosis of liver cancer cells. Nevertheless, several issues in the paper are worthy of attention and comment.

In the paper, echinacoside suppressed the invasion and migration of liver cancer cells after incubation with the agent (10 mg/ml and 20 mg/ml) for 24 h (seeing Fig. 3). However, authors also suggested that echinacoside significantly inhibited cell proliferation and promoted apoptosis of liver cancer cells after incubation with the same working concentration (10 mg/ml and 20 mg/ml) for the same time (24 h) (seeing Fig. 2 and 4). Another two studies also demonstrated that echinacoside could suppress proliferation and induce apoptosis in hepatocellular carcinoma cell line HepG2 at 0.05 mg/ml for 48 h and colorectal cancer cells [2, 3]. These data forcefully showed that the reason responsible for decrease of migration and invasion of liver cancer cells could be owing to apoptosis induction of echinacoside. Authors could treat the cells with echinacoside with lower concentrations or for a short time when investigating the inherent effects of agent on the migration and invasion ability of cells without apoptosis induction. This would make the results more convincing. In addition, as showed in the Fig. 2, there was a significantly difference between 5 mg/ml group and the control group. However, there was no *p* value on the graph. These made us confused.

## Data Availability

Not applicable.
